# Parameter variation effects on millimeter wave dosimetry based on precise skin thickness in real rats

**DOI:** 10.1038/s41598-023-44572-y

**Published:** 2023-10-13

**Authors:** Kun Li, Takashi Hikage, Hiroshi Masuda, Etsuko Ijima, Akiko Nagai, Kenji Taguchi

**Affiliations:** 1 Advanced Wireless and Communication Research Center, The University of Electro-Communications, 182-8585 Tokyo, Japan; 2 Faculty of Information Science and Technology, Hokkaido University, 060-0808 Hokkaido, Japan; 3 Department of Environmental Medicine, Kurume University School of Medicine, Fukuoka, 830-0011 Japan; 4 Department of Anatomy, Aichi-Gakuin University School of Dentistry, Nagoya, 464-8650 Japan; 5 Department of Electrical and Electronic Engineering, Kitami Institute of Technology, Kitami, 090-8507 Japan

**Keywords:** Biomedical engineering, Electrical and electronic engineering

## Abstract

This study presents a parametric analysis of the steady-state temperature elevation in rat skin models due to millimeter wave exposure at frequencies from 6–100 GHz. The statistical data of the thickness of skin layers, namely epidermis, dermis, dermal white adipose tissue, and panniculus carnosus, were measured for the first time using the excised tissues of real male Sprague–Dawley rats. Based on the precise structure obtained from the histological analysis of rat skin, we solve the bioheat transfer equation to investigate the effects of changes in parameters, such as body parts and thermal constants, on the absorbed power density and temperature elevation of biological tissues. Owing to the notably thin dermal white adipose tissue layer, the surface temperature elevation in the rat head and dorsal skin at 6–100 GHz is 52.6–32.3% and 83.3–58.8% of the average values of different human skin models, respectively. Our results also reveal that the surface temperature elevation of rat skin may correlate with the tissue thickness and deep blood perfusion rates.

## Introduction

Rapid growth in the demand for wireless technology employing millimeter wave (MMW) has raised extensive concerns on the safety of human exposure to the electromagnetic fields (EMFs)^1,2^. Unlike the microwave band, in the frequency range of 30–300 GHz, the primary cause of adverse health effects is the superficial heating of the biological tissues owing to the very shallow penetration depths within approximately 1 mm^3–5^. The IEEE International Committee on Electromagnetic Safety (ICES) and the International Commission on Non-Ionizing Radiation Protection (ICNIRP) have revised the exposure standards and guidelines in 2019 and 2020, respectively^6,7^. These documents aim to stipulate the limits for humans in restricted environments/occupational exposure and in unrestricted environments/general public exposure conditions. The present ICNIRP guidelines consider the EMF exposure that results in local temperatures above 41 $^\circ$C is associated with a potential adverse health effects. For different parts of the human body, ICNIRP defines two representive tissue types according to their temperature under normothermal conditions: “Type-1” tissues such as the epidermal, dermal, fat, muscle, and bone tissue; “Type-2” tissues in the head, eye, and abdomen excluding those in Type-1 tissue^7^. The operational thresholds for local heat-induced adverse health effects have been conservatively determined that the temperature elevations should not exceed 5 $^\circ$C and 2 $^\circ$C within Type-1 and Type-2 tissues, respectively^8–10^.

In the latest guidelines, the absorbed power density (APD) within the human skin tissue is recommended as a new internal metric to prescribe the basic restrictions (BRs) of EMF exposures at frequencies from 6–300 GHz. This is because the APD provides a measure of the power absorbed in the tissue that closely approximates the superficial heating in these frequency bands in comparison with the traditional metrics, such as the specific absorption rate (SAR) and incident power density^11–17^. The ICNIRP-2020 guidelines set an APD of 100 W/m$^2$ as the BR value for occupational exposure scenarios for local exposure above 6 GHz in order to provide adequate protection against harmful levels of electromagnetic exposure, i.e., local temperature rise. Furthermore, the BR value for the general public is APD of 20 W/m$^2$, which is the occupational value divided by a reduction factor of 2 (approximately 3 dB).

**Figure 1:**
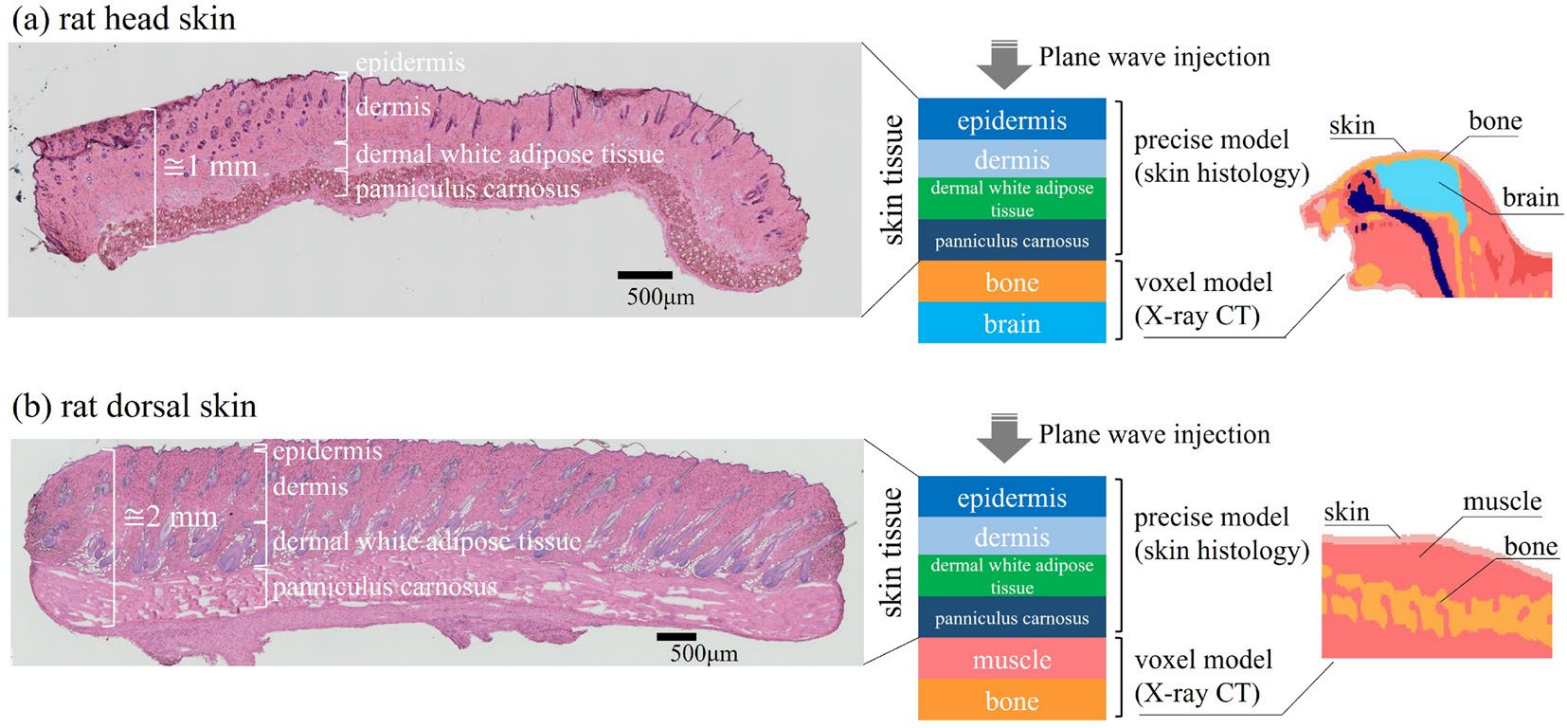
Multi-layer analytical model derived from the cross section of excised rat skin tissue and voxelized model for different body parts: (**a**) rat head, (**b**) rat dorsal.

**Table 1:** Thickness of tissues based on different measurement methods. Three measurements were taken for each stained section of rat skin.

Methods	Histological measurement ($\mu$m)				X-ray CT measurement (mm)		Total thickness (mm)
Body parts	Epidermis	Dermis	dWAT	Panniculus carnosus	Bone/Muscle	Brain/Bone	
	($i=1$)	($i=2$)	($i=3$)	($i=4$)	($i=5$)	($i=6$)	
Head	22.8 ± 2.4	563.4 ± 62.8	191.5 ± 28.0	230.1 ± 25.3	1.0	9.5	11.508
Dorsal	26.1 ± 2.6	908.0 ± 22.4	577.3 ± 45.0	567.5 ± 60.7	4.75	4.75	11.579

Exposure experiments using laboratory animals are a commonly used alternative to obtain the localized temperature elevation in biological tissues^18–20^. Animal studies provide an effective method to examine the thermal effects in animals and extrapolate the findings to people, primarily for ethical reasons concerning health risks to human subjects. Until now, whole-body SAR limits are primarily based on small animal experiments. According to ICNIRP-1998, small animals exposed to microwave bands at and above 4 W/kg performed a characteristic pattern of thermoregulatory behavior^21^. At SAR values ranging from 1–3 W/kg, several experiments using rats and monkeys have shown a decreased task performance^22–24^. However, only limited groups have provided the data of using laboratory animals exposed to MMW^8,9^ due to the lack of exposure equipments. Therefore, numerical analysis methods such as finite-difference time-domain (FDTD), have been widely used in place of real animals to examine the thermal modeling in local MMW exposures^25–30^. Sasaki et al.^31^ investigated the dosimetry using a localized exposure system in the MMW bands for an in vivo study on ocular effects of rabbits. The method has the advantages of being able to reconstruct the internal field inside the voxel model of a rabbit by using a hybrid technique of numerical simulation and measured radiation field of exposure source. Fall et al.^32^ developed an MMW reverberation chamber system to create a statistical multipath exposure environment for animal study, where dosimetry was performed using a mice phantom instead of real animals. These studies did not address dosimetric effects arising from the detailed skin structure of real small animals, primarily because the FDTD simulation cannot satisfy such a high numerical resolution, which may require large amount of computational resources. As the scientific evidence of non-ionizing radiation exposure standards, accurate analysis of thermal effects, i.e., heat conduction among multi-layer skin tissues, is required, it is indispensable to obtain the APD and temperature elevation using a precise model based on realistic skin tissues of laboratory animals.

Recently, Hikage et al.^33^ developed a novel equipment to examine the biological effects of local exposure of the human body to 5G-MMW. The designed system could deliver high-intensity 60 GHz irradiation to the target area of a rat using a spatial synthetic beam exposure setup realized by two dielectric lens antennas. To provide a theoretical basis for dosimetry using the exposure system, this study presents a parametric analysis of the steady-state skin temperature elevation in precise rat models under MMW exposure conditions at 6–100 GHz. To the best of authors’ knowledge, this is the first study that considers a detailed skin model for dosimetry analysis based on excised tissue of real male rats. The behaviors of the APD and skin temperature elevation induced by different exposure conditions and thermal parameters using the developed rat skin model are investigated. The obtained results are important for specifying dosimetric quantities in the case of animal studies at frequencies over 6 GHz, and generalizing the results to humans protection from excessive temperature elevation.

**Figure 2:**
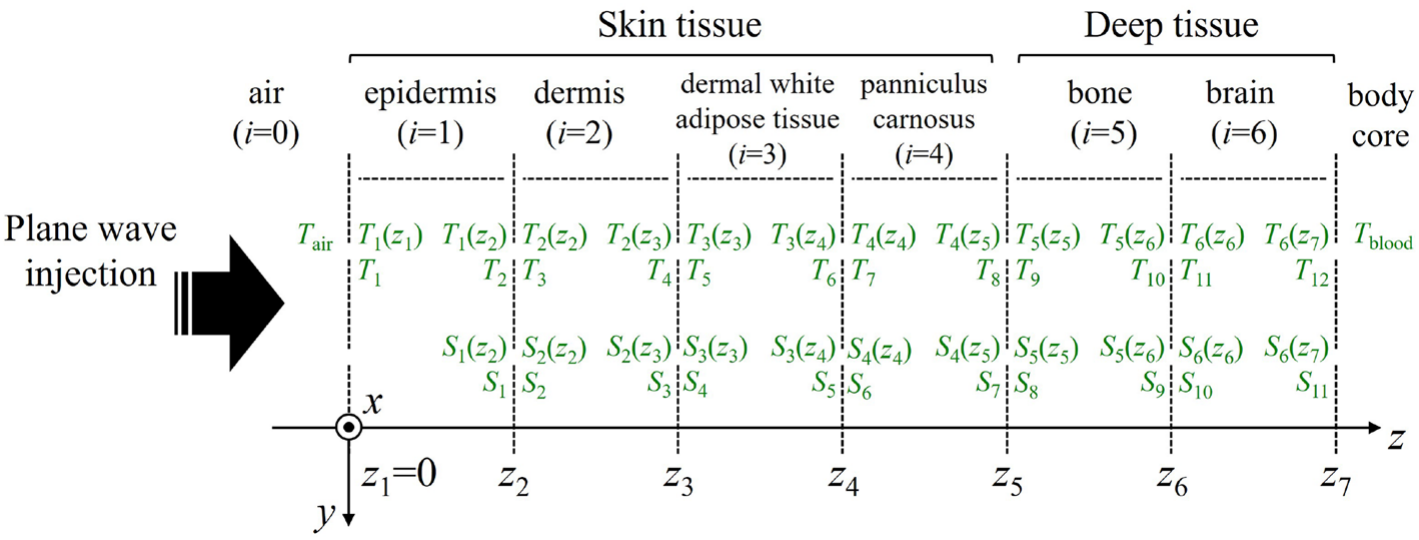
Six-layer model for dosimetry analysis of rat skin at MMWs.

**Table 2:** Thermal parameters employed in the analysis of skin temperature elevation.

Parameters	Epidermis	Dermis	dWAT	Panniculus carnosus	Bone/Muscle	Brain/Bone
	($i=1$)	($i=2$)	($i=3$)	($i=4$)	($i=5$)	($i=6$)
$\kappa _i$ (W/(m$^\circ$C))	0.42	0.42	0.25	0.50	0.37/0.50	0.57/0.37
$\rho _i$ (kg/m$^3$)	1109	1109	911	1090	1990/1090	1038/1990
$A_i$ (W/m$^3$)	7486	7486	4063	1858	7945/1858	10837/7945
$B_i$ (W/(m$^3$ $^\circ$C))	0	11605	6299	380	12317/380	16800/12317

## Method

### Skin thickness analysis

Figure 1 shows the derivation process of a multi-layer analytical model for different body parts on the head and dorsal of a rat. The upper part of the model is based on the precise tissue thickness extracted from the raw skin specimen, i.e., anatomical cross section of real skin tissue in rats, which is composed of the epidermis (${ i}$ = 1), dermis (${ i}$ = 2), dermal white adipose tissue (dWAT) (${ i}$ = 3), and panniculus carnosus (${ i}$ = 4). The lower part corresponds to the voxelized numerical model developed according to the measured X-ray CT images^34^, representing the internal configurations under the skin layers of the rat’s head and dorsal, that is comprised by bone (${ i}$ = 5) and brain layers (${ i}$ = 6) , muscle (${ i}$ = 5) and bone layers (${ i}$ = 6), respectively. Table 1 lists the mean values and standard deviation of tissue thickness. In experiment, eight male Sprague-Dawley rats (8 weeks old, Japan SLC, Japan) were used for histologic analysis of the skin. Anesthetized with with isoflurane (3%<), the rats were sacrificed after shaving the head and dorsal hairs. Skin tissues were excised in 10-mm squares from the head and dorsal area, and immediately frozen with Tissue-Tek®O.C.T. Compound (Sakura Finetek Japan), and then stored at $-$80 $^\circ$C. Cryosections were prepared by cutting at 12 $\mu$m and staining with hematoxylin and eosin. The stained sections were air-dried to prevent shrinkage by dehydration with alcohol. The thickness of each skin layer was measured at three arbitrary areas in each section. Thickness was calculated as mean ± S.D. for four rats for the head and another four rats for the dorsal, respectively. All experimental procedures were performed in accordance with the ethical guidelines for animal experimentation at Kurume University School of Medicine (Approval numbers: 2020-174, 2021-150, and 2022-114). In this study, the measured mean values of the thickness in skin layers were used for dosimetry analysis. In contrast, tissue thickness under the skin layers was determined using a voxelized rat model with a resolution of 0.125 mm. These layers are indispensable because the total thickness of the skin tissue is only within 1–2 mm (see Fig. 1), which is insufficiently deep to ensure the calculation accuracy of heat conduction from the air to the body core temperature.

### Dosimetry analysis

Figure 2 shows a multilayer plane model for dosimetric analysis. A plane wave injected from air to the skin surface is assumed. The incident power density (IPD) is defined at the plane whose normal is parallel to the wave-number $k_0$. The steady-state temperature attributable to the power absorption of injected EM-field was calculated by solving Pennes’s bioheat transfer equation^35–39^

1 $$\begin{aligned} -\frac{\partial }{\partial z} \left( -\kappa _i \frac{\partial T_i(z)}{\partial z} \right) + \rho _i \textrm{SAR}_i(z) +A_i-B_i \left( T_i(z)-T_{\textrm{blood}} \right) = 0, \end{aligned}$$

where $T_i$ denotes the temperature of the skin layers ($^\circ$C). $\kappa _i$ and $\rho _i$ are the thermal conductivity (W/(m$^\circ$C)) and mass density (kg/m$^3$) of *i*-th layer, respectively. $T_{\textrm{blood}}$ is the blood temperature ($^\circ$C). $A_i$ denotes the basal metabolism per unit volume (W/m$^3$). $B_i$ represents a term related with the blood perfusion rate (W/(m$^{3\circ }$C)). The $\textrm{SAR}_{i}$ is the specific absorption rate (W/kg). *i* denotes the tissue ID, as shown in Fig. 2.

The boundary condition that describes the heat conduction between the air and the skin tissue interface for Eq. (1) is expressed as

2 $$\begin{aligned} h\left( T_{1}(z_1)-T_{\textrm{air}}\right) =\kappa _1 \frac{\partial T_1 (z)}{\partial z}|_{z=z_1}, \end{aligned}$$

where *h* is the heat transfer coefficient (W/(m$^{2\circ }$C) between the air and the skin surface. $T_{air}$ denotes the air temperature ($^\circ$C).

Then, the analytical formulae of *T*(*z*) with and without considering the blood circulation phenomenon in the range of $z_i \le z \le z_{i+1}$ are as follows:

- $$B_i=0 \;(i=1)$$

3 $$\begin{aligned} T(z) = T(z_i)+(z-z_i) \frac{\partial T_i (z)}{\partial z}|_{z=z_i} +S_i(z), \end{aligned}$$

- $$B_i>0 \; (i>1)$$

4 $$\begin{aligned} T(z) =\,&T(z_i){\cosh } \sqrt{\frac{B_i}{\kappa _i}} (z-z_i) + \sqrt{\frac{\kappa _i}{B_i}} \sinh \sqrt{\frac{B_i}{\kappa _i}} (z-z_i) \frac{\partial T_i (z)}{\partial z}|_{z=z_i} \nonumber \\&\quad + (T_{\textrm{blood}} + {\frac{A_i}{B_i}} ) \left( 1- {\cosh } \sqrt{\frac{B_i}{\kappa _i}} (z-z_i) \right) +S_i(z), \end{aligned}$$

where $S_i(z)$ is a term that determined by the power absorption of electromagnetic waves. By applying Laplace transform to (3) and (4), and solving them for $\hat{T}(s)=\mathscr {L}[T(z)]$, then $S_i(z)$ can be defined using inverse Laplace transform as follows:

- $$B_i=0 \;(i=1)$$

5 $$\begin{aligned} S_{i,}(z)&=\mathscr {L}^{-1} \left[ {\frac{1}{\kappa _i s^2}} \mathscr {L}[\rho _i {\textrm{SAR}}_{ i,}(z)] \right] \nonumber \\&={\frac{\sigma _i p \eta _0}{\kappa _i}} \left( |\tau _{i,}|^2 (-{\frac{1}{4\alpha _i}} +{\frac{z-z_i}{2\alpha _i}} + {\frac{1}{4\alpha _i}} e^{-2\alpha _i(z-z_i) }\right) + |\upsilon _{i,}|^2 \left( -{\frac{1}{4\alpha _i}} - {\frac{z-z_i}{2\alpha _i}} + {\frac{1}{4\alpha _i}} e^{2\alpha _i(z-z_i) } \right) \nonumber \\&\quad + {\frac{X_{i,}}{2\beta _i}} (1-\cos (2\beta _i (z-z_i) )) + {\frac{Y_{i,}}{2\beta _i}} ( 2\beta _i (z-z_i)-\sin (2\beta _i (z-z_i) ) ) ), \end{aligned}$$

- $$B_i>0 \; (i>1)$$

6 $$\begin{aligned} S_{i,}(z)&= \frac{\sigma _i p \eta _0}{4 \alpha _i^2 \kappa _i -B_i} \left( |\tau _{i,}|^2 (-2\alpha _i \sqrt{\frac{\kappa _i}{B_i}} \sinh \sqrt{\frac{B_i}{\kappa _i}}(z-z_i) + \cosh \sqrt{\frac{B_i}{\kappa _i}} (z-z_i) - e^{-2\alpha _i(z-z_i) } \right) \nonumber \\&\quad + |\upsilon _{i,}|^2 \left( 2\alpha _i \sqrt{\frac{\kappa _i}{B_i}} \sinh \sqrt{\frac{B_i}{\kappa _i}}(z-z_i) + \cosh \sqrt{\frac{B_i}{\kappa _i}} (z-z_i) - e^{2\alpha _i(z-z_i) })\right) \nonumber \\&\quad + \frac{\sigma _i p \eta _0 X_{i,} }{4 \beta _i^2 \kappa _i +B_i} \left( -e^{-\sqrt{\frac{B_i}{\kappa _i}}(z-z_i)} - e^{\sqrt{\frac{B_i}{\kappa _i}}(z-z_i)} + 2\cos 2\beta _i (z-z_i)\right) \nonumber \\&\quad + \frac{2\sigma _i p \eta _0 B_i Y_{i,} }{4 \beta _i^2 \kappa _i +B_i} \left( \sqrt{\frac{\kappa _i}{B_i}}e^{\sqrt{\frac{B_i}{\kappa _i}}(z-z_i)} - \sqrt{\frac{\kappa _i}{B_i}}e^{\sqrt{\frac{B_i}{\kappa _i}}(z-z_i)} + \frac{\sin 2\beta _i (z-z_i)}{\beta _i}\right) , \end{aligned}$$

where *p* represents the IPD (W/m$^2$) outside the body. $\eta _i$, $\tau _{i}$, and $\upsilon _{i}$ denote the wave impedance, transmission coefficient, and reflection coefficient at the boundary interface of the *i*-th layer, respectively. $\beta _i$ and $\alpha _i$ denote the phase and attenuation constant in each layer in the direction normal to the skin model.

By solving the boundary conditions for the heat conduction in each layer, the temperature distribution along the *z*-axis is calculated by the following matrix equation:

7 $$\begin{aligned}&\left( \begin{array}{cccccccccccc} 1 &{} {\frac{\kappa _3}{h}} &{}0 &{}0 &{}\dots &{}0 &{}0 &{}0 \\ 1 &{} d_2 &{}-1 &{}0 &{}\dots &{}0 &{}0 &{}0\\ 0 &{} 1 &{}0 &{}-{\frac{\kappa _3}{\kappa _2}} &{}\dots &{}0 &{}0 &{}0\\ 0 &{} 0 &{}\cosh \sqrt{\frac{B_3}{\kappa _3}}d_3 &{} \sqrt{\frac{\kappa _3}{B_3}} \sinh \sqrt{\frac{B_3}{\kappa _3}}d_3 &{}\dots &{}0 &{}0 &{}0\\ \vdots &{}\vdots &{}\vdots &{}\vdots &{}\ddots &{}\vdots &{}\vdots &{}\vdots \\ 0 &{} 0 &{}0 &{}0 &{}\dots &{}\sqrt{\frac{\kappa _6}{B_6}} \sinh \sqrt{\frac{B_6}{\kappa _6}}d_6 &{}-1 &{}0\\ 0 &{} 0 &{}0 &{}0 &{}\dots &{}\cosh \sqrt{\frac{B_6}{\kappa _6}}d_6 &{}0 &{}-{\frac{\kappa _7}{\kappa _6}} \\ 0 &{} 0 &{}0 &{}0 &{}\dots &{}0 &{}\cosh \sqrt{\frac{B_7}{\kappa _7}}d_7 &{}\sqrt{\frac{\kappa _7}{B_7}} \sinh \sqrt{\frac{B_7}{\kappa _7}}d_7 \end{array}\right) \nonumber \\&\cdot \left( \begin{array}{c} T_1 \\ T_2 \\ T_3 \\ T_4 \\ \vdots \\ T_{10} \\ T_{11} \\ T_{12} \end{array}\right) =\left( \begin{array}{c} T_{\textrm{air}} \\ {\frac{A_2}{2\kappa _2}}d_2^2 - S_1+S_2 \\ {\frac{A_2}{\kappa _2}} d_2^2 - S_1'+ {\frac{\kappa _3}{\kappa _2}}S_2' \\ -(T_{\textrm{blood}}+{\frac{A_3}{B_3}})\cdot (1-\cosh \sqrt{\frac{B_3}{\kappa _3}}d_3)-S_3+S_4 \\ \vdots \\ -(T_{\textrm{blood}}+{\frac{A_6}{B_6}})\cdot (1-\cosh \sqrt{\frac{B_6}{\kappa _6}}d_6)-S_9+S_{10} \\ \sqrt{\frac{B_6}{\kappa _6}} (T_{\textrm{blood}}+{\frac{A_6}{B_6}}) \sinh \sqrt{\frac{B_6}{\kappa _6}}d_6-S_9'+{\frac{\kappa _7}{\kappa _6}}S_{10}' \\ T_{\textrm{body}}-(T_{\textrm{blood}}+{\frac{A_7}{B_7}})\cdot (1-\cosh \sqrt{\frac{B_7}{\kappa _7}}d_7 )-S_{11} \end{array}\right) . \end{aligned}$$

In Eq. (7), $d_i$ indicates the thickness of *i*th layer derived by $z_{i+1}-z_i$. $T_{\textrm{body}}$ is the body core temperature. $S_i'$ denotes the differential expression of $S_i$.

For consistency with the results obtained by previous studies^25,36,37^, the similar thermal parameters were set in the temperature elevation analysis and are listed in Table 2. Note that in the actual experiment, the thermal parameters may be affected by several factors, such as different animals and different measurement environments. The heat transfer coefficient between the air and the rat skin surface was set to 0.5 W/(m$^{2\circ }$C)^25,26^. The temperature at the end of the skin model was fixed as the body core temperature (37 $^\circ$C). $T_{\textrm{blood}}$ and $T_{\textrm{air}}$ were set to 37 $^\circ$C and 23 $^\circ$C, respectively. The dielectric characteristics of skin tissues reported by Sasaki et al.^36^ were used, whereas for deep tissues, we employed the databases developed by Gabriel et al.^40^. The temperature elevation was obtained for the difference between the exposure to EMFs and that before exposure.

## Results

### APD vs. IPD

**Figure 3:**
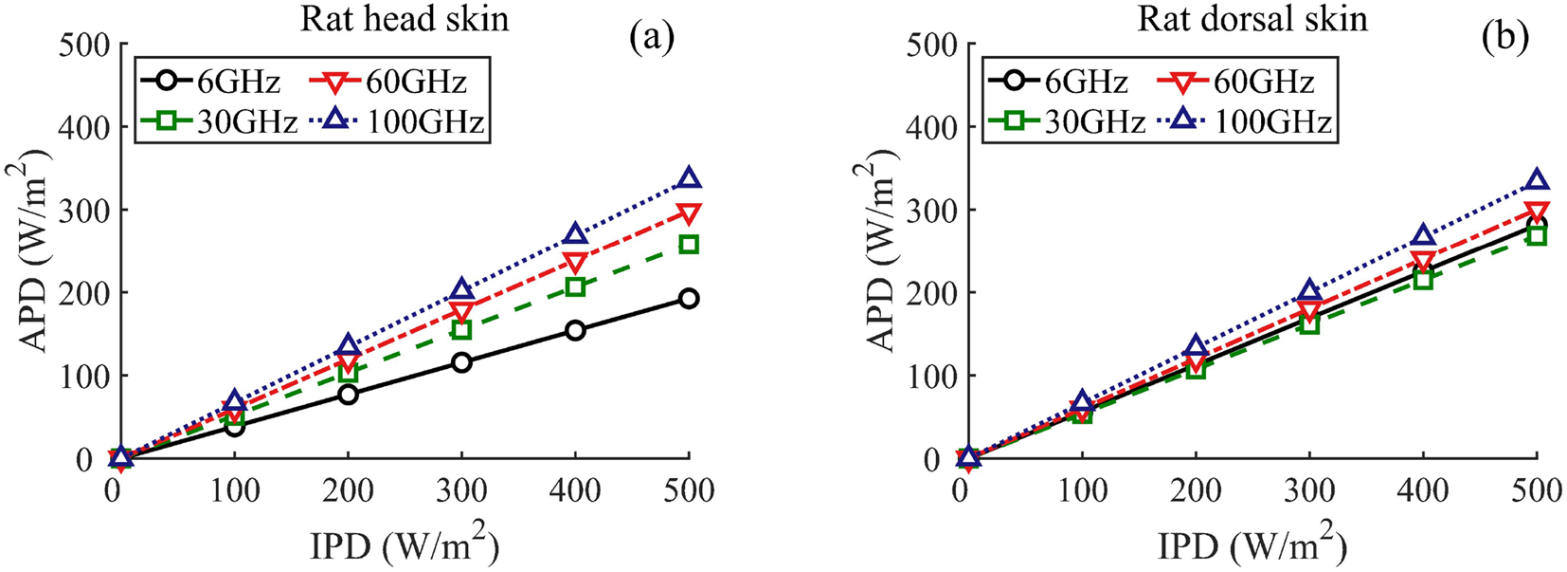
APD at the skin surface versus IPD outside the body at 6, 30, 60, and 100 GHz, (**a**) rat head, (**b**) rat dorsal.

Figure 3 shows the linear relationship between the APD at the skin surface and the IPD outside the body at frequencies of 6, 30, 60, and 100 GHz, respectively. Fig. 3a and b indicate the results for different body parts: the rat’s head and dorsal skin, respectively. The APD at the skin surface is defined as follows:

8 $$\begin{aligned} APD=\frac{1}{2} \mathfrak {Re} \left[ \left( \textbf{E}(z) \times \textbf{H}^{*}(z) \right) \cdot n \right] |_{z=z_1}, \end{aligned}$$

where $\textbf{E}$ and $\textbf{H}^{*}$ indicate the electric-field phasor and the complex conjugate of the magnetic-field phasor inside the biological tissues, respectively. *n* denotes the unit vector normal to the skin model. Note that the IPD represents the power crossing a unit area, with its normal oriented in the direction of the wave vector of the incident wave. For the normal incidence exposure scenario examined in this study, the ratio of APD to IPD, which corresponds to the slope of each line in Fig. 3, indicates the transmittance at the skin surface^37^.

As shown in Fig. 3a, for the rat head skin, there is a noticeable deviation in the slope between the lines. The transmittance increases from 0.38 to 0.67 over the frequency range of 6–100 GHz. In the case of the rat’s dorsal skin, as illustrated in Fig. 3b, the transmittance shifts from 0.56 to 0.67 from 6–100 GHz. In comparison to the head skin tissue, the dorsal skin of the rat exhibits a narrower range of transmittance variation at the skin surface relative to frequency.

### Temperature elevation vs. APD

**Figure 4:**
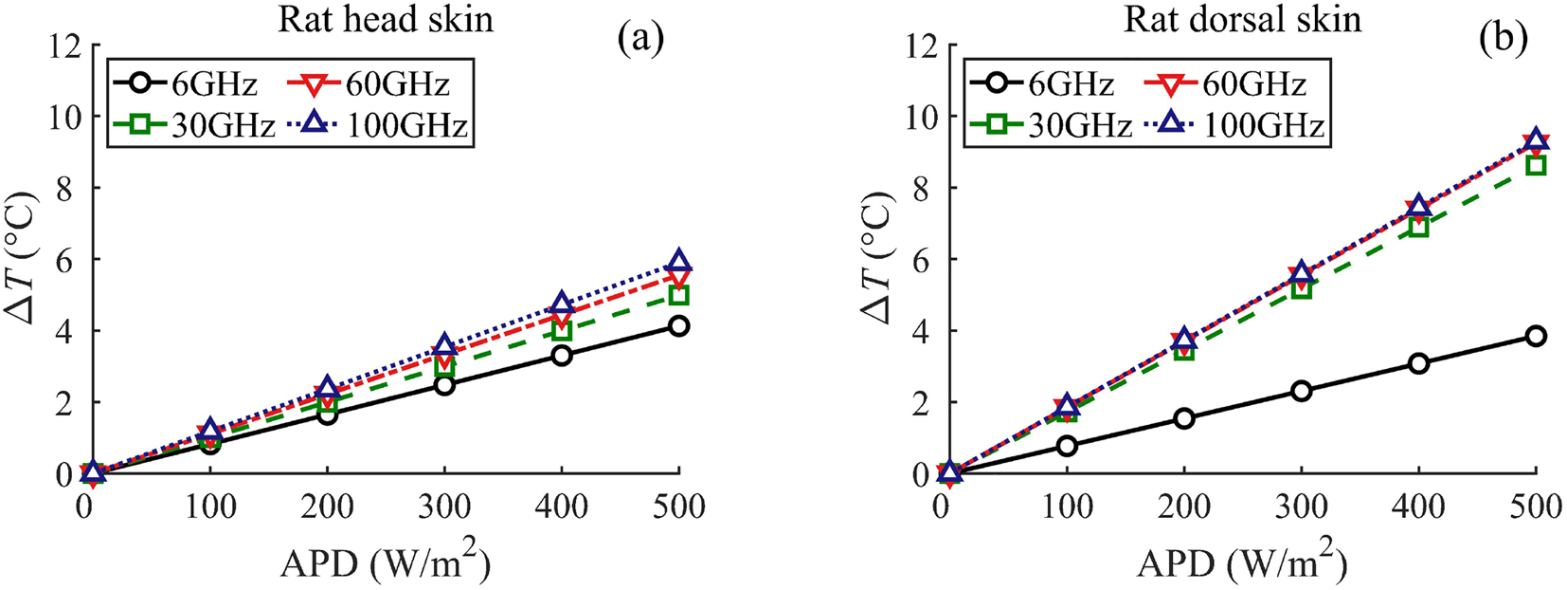
$\Delta { T}$ versus APD at the skin surface at 6, 30, 60, and 100 GHz, (**a**) rat head, (**b**) rat dorsal.

Figure 4 shows the linear relationship between the skin surface temperature elevation ($\Delta { T}$) and the APD at frequencies of 6, 30, 60, and 100 GHz, respectively. The slope of each line in the figure, i.e., the ratio of the $\Delta { T}$ to the APD at the skin surface, represents the heating factor. This metric is widely-used in MMW dosimetry analysis to estimate the peak steady-state temperature elevation by multiplying the corresponding values to the exposure limit^12,41^.

In Fig. 4a, the heating factor for the rat head skin varies from 0.0083 to 0.0118 $^{\circ }$C m$^2$/W over the range of 6–100 GHz. Conversely, for the rat dorsal skin, as illustrated in Fig. 4b, the heating factor is 0.0077 $^{\circ }$C m$^2$/W and ranges from 0.0172 to 0.0186 $^{\circ }$C m$^2$/W between 30–100 GHz. The results indicate that, above 30 GHz, the equivalent level of APD might cause a 70% higher $\Delta { T}$ in the rat dorsal skin compared to the rat head skin. Additionally, the analytical findings in Fig. 4b align well with the experimental investigation using a real rat (see Fig. 6 in^42^), demonstrating the effectiveness of dosimetry analysis with the precise rat skin model.

### APD and temperature elevation vs. depth direction in skin

**Figure 5:**
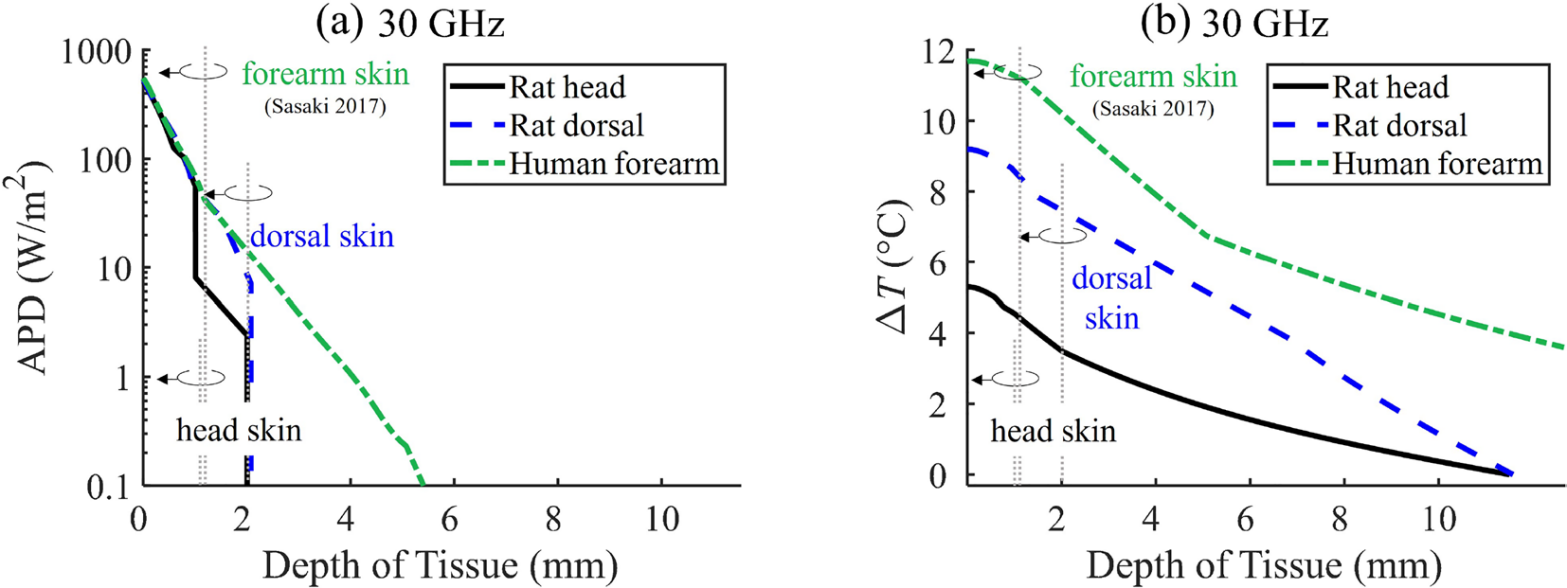
Comparison of rat head and dorsal with human forearm skin as a function of the depth of tissue at 30 GHz when IPD is normalized to 1000 W/m$^2$: (**a**) APD, (**b**) $\Delta { T}$.

Figure 5a and b illustrate the distribution of the APD and the $\Delta { T}$ in the depth direction of the skin tissue at 30 GHz, respectively. For comparison, we used the four layer human forearm skin model presented by Sasaki et al. in 2017^36^. The IPD was normalized to 1000 W/m$^2$.

In Fig. 5a, it can be seen that there is no significant difference in the APD at the skin surface among the rat head, rat dorsal, and human forearm skin models. As tissue depth increases, a pronounced drop in the APD occurs at $z \approx$ 1 mm and 2 mm for the rat head and dorsal skin models, respectively. In contrast, the human forearm skin contains thicker subcutaneous fat and muscle layers, totaling more than 27 mm. This means the rate of decrease in APD with respect to tissue depth is more gradual compared to the rat skin models. The power density is primarily absorbed within the skin layers of both the rat head and dorsal, which are $\approx$1 mm and $\approx$2 mm thick, respectively.

In Fig. 5b, a distinct difference is observed in the $\Delta { T}$ distributions among the rat head, rat dorsal, and human forearm skin models. Within the skin layers of the rat’s head, $\Delta { T}$ decreases by 14.1% compared to the skin surface. In the cases of the rat’s dorsal skin, the corresponding reduction is 19.4%. The difference in $\Delta { T}$ within the upper skin layers, which elicits warmth sensations in both the rat’s head and dorsal, ranges from 54.7 to 66.2%. One potential explanation for these results is that the skin thickness on the rat’s dorsal is almost double that of its head and includes a thicker dWAT layer, which might provide enhanced adiabatic effects. On the other hand, due to its relatively thick subcutaneous fat and muscle layers, the human forearm skin model displays a higher $\Delta { T}$ distribution than the rat skin models. These findings suggest that $\Delta { T}$ in the tissue depth direction is heavily influenced by the structure of the skin model.

**Figure 6:**
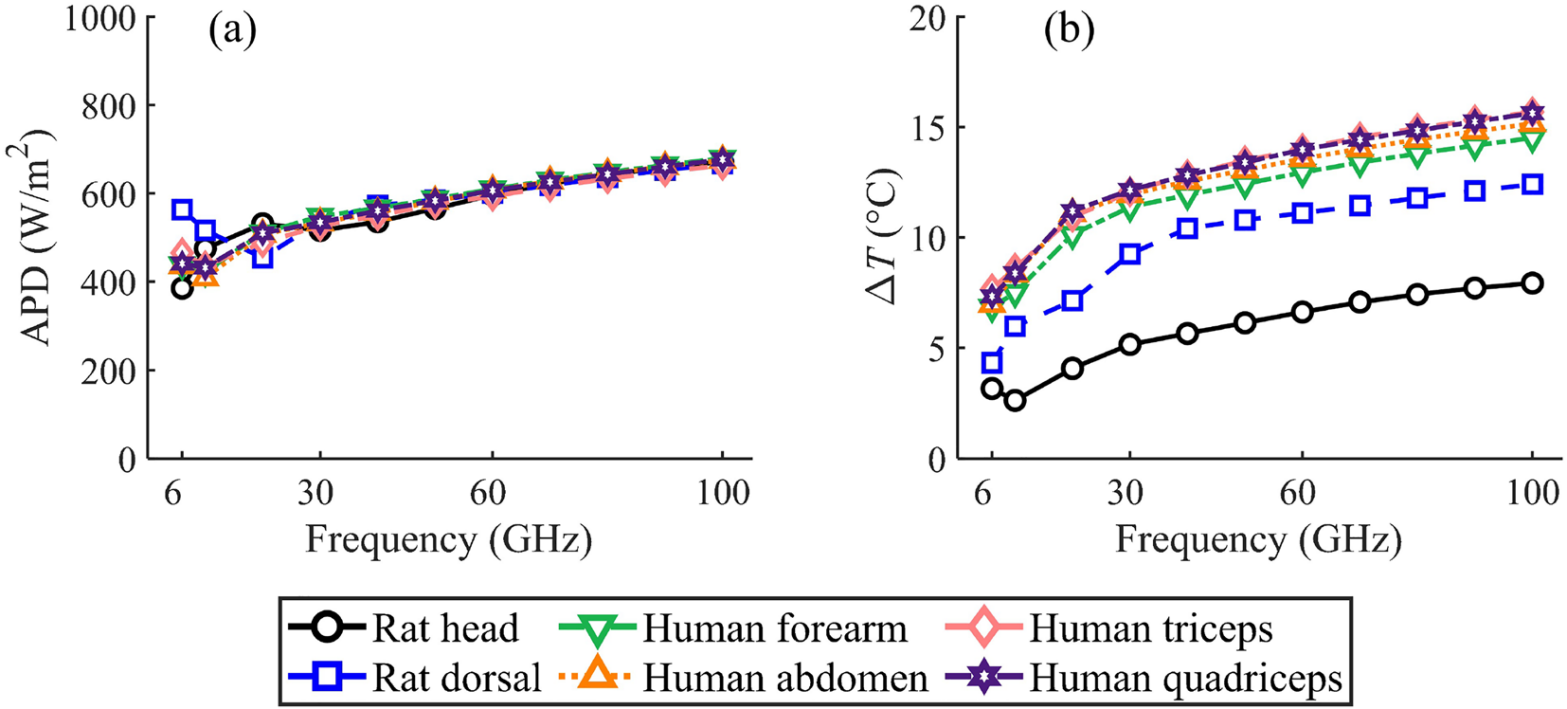
Variations as a function of frequency from 6–100 GHz using different body parts of rat and human skin when IPD is normalized to 1000 W/m$^2$: (**a**) APD, (**b**) $\Delta { T}$.

### APD and temperature elevation vs. frequency

Fig. 6a and b show the APD and $\Delta { T}$ at the skin surface for frequencies ranging from 6–100 GHz, respectively. In addition to the rat head and dorsal skin, the results include data from human skin tissue atthe forearm, abodomen, triceps, and quadriceps for comparison. The skin tissue thickness for different human body parts from previous study was used (see Table 2 in^36^).

As depicted in Fig. 6a, the APD at skin surface coincides well between the rat and human skin models. The relative standard deviation (RSD) among the APDs of different skin models remains within 13% for frequencies ranging from 6 to 30 GHz, and does not exceed 2.4% for frequencies above 30 GHz. Under identical exposure levels of the IPD, the power absorbed by both the rat and the human body shows minimal variance with the considered MMW bands. This observation suggests that the structure of the skin models has a minimal effect on the APD at the skin surface. This minimal variance is attributed to the fact that, at MMWs, the APD at the skin surface is largely determined by its reflection coefficient, i.e., $\upsilon _0$, at the air-to-skin boundary interface.

On the other hand, a significant difference in $\Delta { T}$ at skin surface is observed between the rat and human skin models. The RSD among the $\Delta { T}$ of different skin models increases to 33.4% at 6–30 GHz and remains above 26.7% at 30–100 GHz. Across the entire frequency range of 6 to 100 GHz, the $\Delta { T}$ for the rat head and dorsal skin are 52.6 to 32.3% and 83.3 to 58.8% of the average values of the four types of human skin models, respectively. This observation implies that the $\Delta { T}$ at the rat skin surface might also be influenced by the thermal parameters in the deeper tissue layers.

**Table 3:** Thermal resistance in the different skin model layers.

Tissue	Mean layer thickness ($\mu$m)	Steady-state temperature rise at top surface of layer $\Delta { T}$ ($^\circ$C)	IPD (W/m$^2$)	APD (W/m$^2$)	Thermal resistance $R_i$ ($^\circ$C m$^2$/W)
(a) Rat head skin					
Epidermis	22.8	5.3		516	0.0001
Dermis	563.4	5.3	1000	494	0.0013
dWAT	191.5	5.0		127	0.0008
Panniculus carnosus	230.1	4.7		103	0.0005
(b) Rat dorsal skin					
Epidermis	26.1	9.2		537	0.0001
Dermis	908	9.2	1000	510	0.0022
dWAT	577.3	8.7		60	0.0023
Panniculus carnosus	567.5	7.8		29	0.0011
(c) Human forearm skin					
Epidermis	102	11.7		548	0.0002
Dermis	1080	11.7	1000	458	0.0026
Subcutaneous fat	3890	11.2		45	0.0156
Muscle	23250	6.8		0.2	0.0465

## Discussion and conclusion

In this section, we discuss the variation of the temperature elevation caused by the structure of skin models and thermal parameters of the blood perfusion rate in each layer.

### Variability of temperature elevation due to model structure

Given that the precise rat skin model proposed in this study differs in thickness and configuration from the conventionally used multi-layer human skin model, it is recommended to analyze this issue from the perspective of thermal resistance^43^.

Table 3 summarizes the tissue thickness and analytical solutions at 30 GHz for the rat head, dorsal, and human forearm skin model, respectively. The IPD was normalized to 1000 W/m$^2$. Assuming the analytical model represents a purely thermally conductive multi-layer slab, the calculation of the thermal resistance ($R_i$) for each layer can be simplified as follows:

9 $$\begin{aligned} R_i=\frac{d_i}{\kappa _i}, \end{aligned}$$

where $d_i$ is the tissue thickness, and $\kappa _i$ is the thermal conductivity. Similar to the outcomes from Fig. 5, as shown in Table 3, the APD at the skin surface across different skin models aligns closely and clearly diminishes with increased tissue depth. However, the $\Delta { T}$ at the top surface of each layer exhibits noticeable discrepancies among the models and decreases more gradually with the increase in tissue depth compared with APD. In terms of thermal resistance, the rat’s epidermis and dermis closely resemble that of the human forearm skin. Yet, due to the exceedingly thin dWAT layer in the rat skin, its thermal resistance on the head and dorsal is 5.1% and 14.7% of the subcutaneous fat layer of the human forearm skin, respectively. Drawing from the research on temperature elevation variations relative to fat tissue thickness conducted by Alekseev and Ziskin et al.^44^, it becomes evident that the thermal resistance effect of the rat’s dWAT layer, essentially its fat tissue, is not as significant as that of the human forearm skin.

### Variability of temperature elevation due to thermal parameters

**Figure 7:**
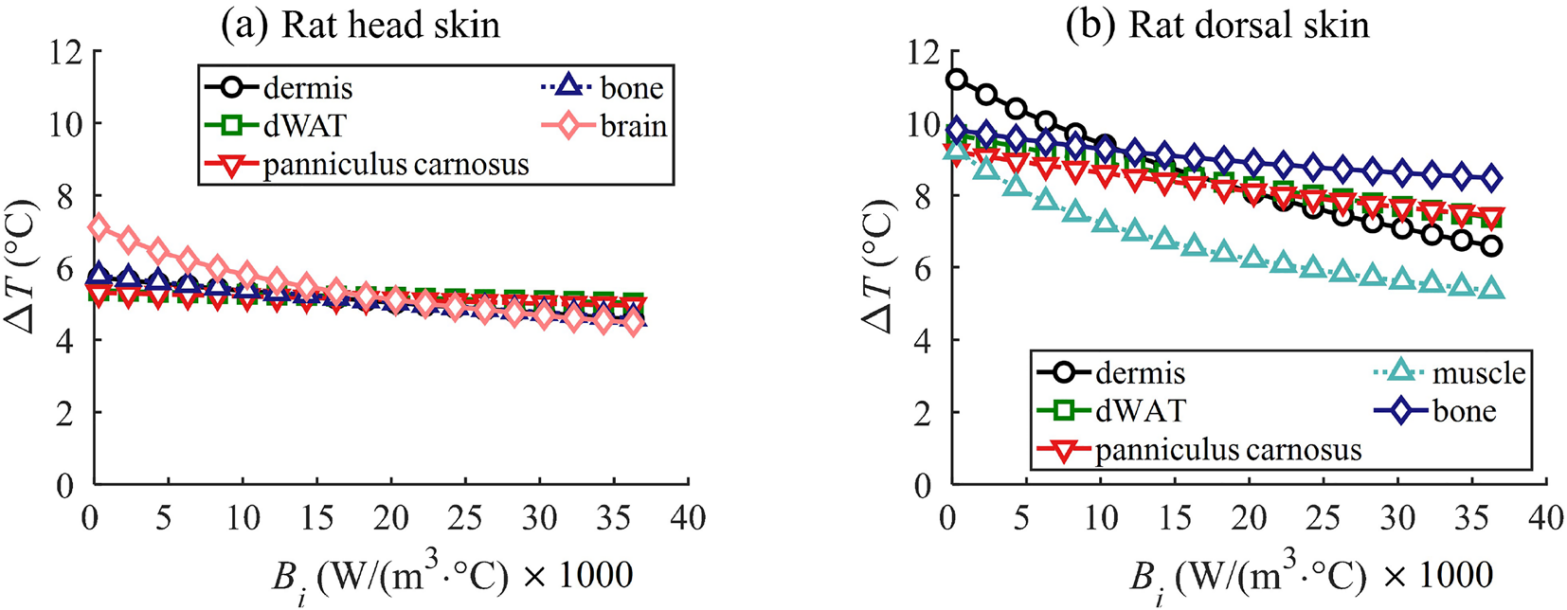
$\Delta { T}$ at skin surface at 30 GHz considering the variation in blood perfusion rate ($B_i$) in each skin layer for different body parts, (**a**) rat head, (**b**) rat dorsal.

Figure 7a and b illustrates the $\Delta { T}$ at the surface of rat’s head and dorsal skin, respectively, as a function of the term of blood perfusion rate $B_i$ across different layers. Note that the epidermis layer has no blood circulation in the considered model, which means $B_1 = 0$. The range of $B_i$ for all layers was adjusted between 300–36300 (W/(m$^3$ $^\circ$C)), drawing from the thermal parameters used in Hirata et al.^25^ and Sachiko et al.^26^. These parameters represent the typical blood perfusion rate for rats aged between 4 and 8 weeks. The analysis was conducted at a frequency of 30 GHz.

In Fig. 7a, for the rat head skin, the $\Delta { T}$ at the skin surface appears largely independent of the blood perfusion rate in most rat skin layers. However, it does exhibit a minor dependence on the deep brain tissue. As the $B_i$ in the brain layer of the rat head skin increases, the $\Delta { T}$ at the skin surface decreases by approximately 37%. For the rat dorsal skin, as depicted in Fig. 7b, the $\Delta { T}$ at the skin surface show the variations in $B_i$ for each skin layer. Notably, the dermis layer experiences a pronounced fluctuation in $\Delta { T}$, with variations up to 41% at the skin surface compared to other layers. As the considered $B_i$ increases, the bone layer in the deeper tissue performs a heightened $\Delta { T}$ at the skin surface. These findings underscore that the $\Delta { T}$ at the skin surface might be more influenced by the model structure of distinct rat body parts than by changes in the blood perfusion rate. Additionally, the blood perfusion rate in deeper tissues seems to notably affect surface temperature elevation in regions like the rat’s head, where the skin is markedly thinner.

In this study, we calculated the absorbed power density and the steady-state temperature elevation in different rat skin models exposed to MMW at 6–100 GHz. The measurement data of the tissue thickness using real rat skin on head and back is given for the first time for dosimetry analysis. By solving the electromagnetic field boundary condition and bioheat transfer equation using the developed model, model structures are known to have no significant effect on APD at the rat skin surface. From analytical solution, it is known that the surface temperature elevation in the rat head and dorsal skin at 6–100 GHz is 52.6–32.3% and 83.3–58.8% of the average values of different human skin models, respectively. Note that the results are based on a model of excised skin tissue obtained after shaving. If the effects of animal fur are considered, the thermal impacts may need to be evaluated separately. On the other hand, the one-dimensional model of rat skin subjected to plane wave illumination in this study does not comprehensively represent the three-dimensional distribution of electromagnetic energy within the body due to exposure from MMW antennas, or the subsequent heat diffusion behavior in a realistic body scenario. This limitation should be explored in our subsequent research. The results also reveal that the skin surface temperature elevation may be correlated with the blood perfusion rates in the deeper layers as well as the thickness of the skin tissues. The results of this study may serve as useful values in the dosimetric evaluation of experiments in laboratory animals, which are expected to be relevant for the protection of humans from excessive local temperature elevation due to MMW exposure.
